# Microaerobic conditions caused the overwhelming dominance of *Acinetobacter* spp. and the marginalization of *Rhodococcus* spp. in diesel fuel/crude oil mixture-amended enrichment cultures

**DOI:** 10.1007/s00203-019-01749-2

**Published:** 2019-10-29

**Authors:** Fruzsina Révész, Perla Abigail Figueroa-Gonzalez, Alexander J. Probst, Balázs Kriszt, Sinchan Banerjee, Sándor Szoboszlay, Gergely Maróti, András Táncsics

**Affiliations:** 1 Regional University Center of Excellence in Environmental Industry, Szent István University, Páter K. u. 1., Gödöllő, 2100 Hungary; 2 Department of Environmental Safety and Ecotoxicology, Szent István University, Gödöllő, Hungary; 3 Biofilm Centre, University of Duisburg-Essen, 45141 Essen, Germany; 4 Institute of Plant Biology, Biological, Research Centre of the Hungarian Academy of Sciences, Szeged, Hungary

**Keywords:** Petroleum hydrocarbons, Biodegradation, Groundwater, Alkane-monooxygenase, alkB, *Rhodococcus*, *Acinetobacter*, *Candidatus* saccharibacteria, Genome-resolved metagenomics

## Abstract

The aim of the present study was to reveal how different microbial communities evolve in diesel fuel/crude oil-contaminated environments under aerobic and microaerobic conditions. To investigate this question, aerobic and microaerobic bacterial enrichments amended with a diesel fuel/crude oil mixture were established and analysed. The representative aerobic enrichment community was dominated by Gammaproteobacteria (64.5%) with high an abundance of Betaproteobacteriales (36.5%), followed by Alphaproteobacteria (8.7%), Actinobacteria (5.6%), and *Candidatus* Saccharibacteria (4.5%). The most abundant alkane monooxygenase (*alkB*) genotypes in this enrichment could be linked to members of the genus *Rhodococcus* and to a novel Gammaproteobacterium, for which we generated a high-quality draft genome using genome-resolved metagenomics of the enrichment culture. Contrarily, in the microaerobic enrichment, Gammaproteobacteria (99%) overwhelmingly dominated the microbial community with a high abundance of the genera *Acinetobacter* (66.3%), *Pseudomonas* (11%) and *Acidovorax* (11%). Under microaerobic conditions, the vast majority of *alkB* gene sequences could be linked to *Pseudomonas veronii*. Consequently, results shed light on the fact that the excellent aliphatic hydrocarbon degrading *Rhodococcus *species favour clear aerobic conditions, while oxygen-limited conditions can facilitate the high abundance of *Acinetobacter *species in aliphatic hydrocarbon-contaminated subsurface environments.

**Electronic supplementary material:**

The online version of this article (10.1007/s00203-019-01749-2) contains supplementary material, which is available to authorized users.

## Introduction

Petroleum hydrocarbons are still among the most common environmental contaminants. Accidental spills and leaks occurring during transport and storage of crude oil and refine products cause the majority of these contaminations. In terrestrial ecosystems, petroleum hydrocarbon pollutions considerably threaten subsurface water reservoirs, which are often the primary sources of drinking water. Soil contaminations can also cause serious damage in the affected ecosystem since the pollutants can accumulate in animal and plant tissues causing death or mutations. Saturated hydrocarbons (alkanes) are quantitatively the most abundant fraction among all petroleum hydrocarbons (Mbadinga et al. 2011). Although alkanes show relatively low reactivity, several microorganisms can use them as a sole source of carbon and energy coupled with the reduction of different electron acceptors (Mbadinga et al. 2011). Nevertheless, the most rapid biodegradation of alkanes can be observed under aerobic conditions due to the fact that under anaerobic conditions the chemical inertness of the carbon–carbon bond retards the degradation (Singh et al. 2012). For this reason, in case of bioremediation projects aiming the cleanup of large-scale terrestrial contaminations (e.g. the cleanup of pollutions at former Soviet military bases in Central and Eastern Europe), aerobic conditions are preferred (Sarlos and Gondár 1995; Kabelitz et al. 2009). However, aeration of such large-scale contaminated sites is a considerably costly and energy-consuming process. In the light of this fact, it would be beneficial to uncover the diversity of those aerobic alkane-degrading microbes, which are adapted to hypoxic/microaerobic conditions. Still, only a handful of studies have dealt with this question to date.

The initial step of aerobic alkane degradation is the incorporation of molecular oxygen into the hydrocarbon molecule by the activity of oxygenases (Mbadinga et al. 2011). Depending on the chain-length of the alkane molecule, different enzymes play a role in their initial oxidation. The C1–C4 alkanes are initially oxidized by methane-monooxygenase-like enzymes, the C5–C16 alkanes by cytochrome P450 or integral membrane non-heme iron (alkB) enzymes, while in case of longer alkanes essentially unknown enzyme systems play a role (van Beilen and Funhoff 2007). Either way, the presence of oxygen is necessary for the activity of these enzymes. Nevertheless, in subsurface ecosystems, the availability of oxygen is often restricted even in pristine environments. On the other hand, hydrocarbons are potential carbon and energy sources for several aerobic microorganisms. Therefore, contamination increases the microbial metabolism and consequently, the aerobic microbial respiration and accompanying biological processes decrease the dissolved oxygen concentration in the contaminated environments (Táncsics et al. 2013). In case of aromatic hydrocarbon degradation, it was observed that distinctly different microbial communities take part in the process under aerobic and oxygen-limited conditions (Martirani-Von Abercron et al. 2017; Benedek et al. 2018). A group of extradiol dioxygenases (in subfamily I.2.C of extradiol dioxygenases) was shown to be adapted to low substrate concentrations, thus function under hypoxic conditions as well (Kukor and Olsen 1996). Recently, it has been shown that Rhodocyclaceae bacteria harboring subfamily I.2.C-type extradiol dioxygenases played a key role in the microaerobic degradation of toluene in a BTEX-contaminated groundwater sediment (Táncsics et al. 2018; Bradford et al. 2018). Recently, a couple of studies have reported on alkane degradation under oxygen-limited conditions or alkane-degrading microbial communities in oxygen-limited environments (e.g. oil reservoirs) (Cyplik et al. 2011; Liu et al. 2018; Tribelli et al. 2018). Still, further research is needed to better understand the effect of decreased oxygen availability on the structure of contaminant-degrading bacterial communities. Thus, the aim of the present study was to reveal how different bacterial communities evolve in diesel fuel/crude oil-contaminated environments under aerobic and microaerobic conditions. Accordingly, aerobic and microaerobic (~ 0.5 g mL^−1^ dissolved oxygen concentration) bacterial enrichments amended with a diesel fuel/crude oil mixture were established and investigated.

## Materials and methods

### Enrichment setup

To reveal the effect of oxygen limitation on the structure of oil-degrading bacterial communities, fully aerobic (~ 7–8 mg L^−1^ O_2_) and microaerobic (≤ 0.5 mg L^−1^ O_2_) enrichment microcosms were set up in triplicates in a defined freshwater medium as described earlier (Benedek et al. 2018). To gain inoculum for enrichment cultures, biofilm sample was collected from groundwater well of a deeply investigated gasoline contaminated site of Hungary, which has already been described in some of our earlier studies (Benedek et al. 2016, 2018). Subsequently, 1 g (wet weight) of biofilm was added to 99 mL of physiological saline solution, and 5 mL of this solution was then used to inoculate each of the first enrichments. Enrichment cultures were set up in 100-mL crimp-sealed serum bottles, which contained 45 mL of the freshwater medium, 5 mL inoculum and 20 ppm diesel fuel/crude oil mixture (3:2, v/v) as a sole source of carbon and energy. Both crude oil and pure (additive-free) diesel fuel were obtained from the Hungarian Oil and Gas Plc. (MOL Plc.) Before inoculation of the microaerobic enrichments, microcosms were sparged aseptically with N_2_/CO_2_ (80:20, v/v) for 10 min. After that, the desired volume of sterile air (0.2-μm-pore-size-filtered) was injected into the bottles through butyl-rubber septa to set the 0.5 mg L^−1^ O_2_ concentration. Dissolved oxygen concentration in the liquid phase of both aerobic and microaerobic enrichments was measured and monitored non-invasively using a Fibox 3 trace v3 fibre optic oxygen meter with PSt3 sensor spots (PreSens). In the case of oxygen depletion, supplementation took place to maintain either clear aerobic or microaerobic conditions. Enrichments were incubated in a rotary incubator (28 °C, 150 rpm) for 1 week, and then 5 mL of each enrichment was transferred to 45 mL of fresh enrichment medium. Transfers were repeated for 5 consecutive weeks.

### DNA isolation and T-RFLP fingerprinting

To isolate DNA from the enrichments, the microbial biomasses were harvested from 45 mL of the enrichments by centrifugation at 2360 *g* at 4 °C for 10 min using a Rotanta 460 R centrifuge (Hettich), and DNA was isolated from the pellets using the DNeasy UltraClean Microbial Kit (Qiagen) according to the instructions of the manufacturer. To isolate DNA from the biofilm inoculum, the NucleoSpin Soil Kit (Macherey–Nagel) was used by following the instructions of the manufacturer. For 16S rDNA-based community profiling, the VIC-labeled amplicons were generated using 27f-VIC (5′-AGA GTT TGA TCM TGG CTC AG-3′) and 1492r primers (5′-TACGGYTACCTTGTTACGACTT-3′), as described earlier (Benedek et al. 2016). For *alkB*-based T-RFLP, the VIC-labeled amplicons were generated using forward primer alkB 1f_deg-VIC (5′-AAY ACI GCI CAY GAR CTI GGI CAY AA-3′) and reverse primer alkB 1r_deg (5′-GCR TGR TGR TCI GAR TGI CGY TG-3′) developed by Kloos et al. (2006) and slightly modified by Pérez-de-Mora et al. (2011). The PCR cycle program was the same as it was described by Giebler et al. (2014). The PCR reaction mixture (final volume of 50 µL) included: 5 µL of 10 × DreamTaq Buffer (Thermo Fisher Scientific), 0.2 mM of each of the four dNTP, 0.3 μM of each primer, 0.25 µL of a 5-U μL^−1^ DreamTaq DNA Polymerase solution (Thermo Fisher Scientific), 20 ng template DNA and autoclaved MilliQ water up to 50 µL. All amplifications were performed in a ProFlex PCR System (Life Technologies). All amplification products were checked by electrophoresis on 1% agarose gels stained with ethidium bromide.

To gain T-RFLP electropherograms, the VIC-labelled 16S rDNA amplicons were digested with *RsaI* (Thermo Fisher Scientific) while the *alkB* amplicons were digested with *HPyCH4V* (New England BioLabs), as described earlier (Révész et al. 2006). After ethanol precipitation, fragments were separated on a Model 3130 Genetic Analyzer (Applied Biosystems), while primary evaluation of electropherograms was performed using GeneMapper 4.0 software (Applied Biosystems). T-RFLP data were handled as described earlier (Farkas et al. 2017). Cluster analysis (Jaccard and Bray–Curtis methods) of the T-RFLP electropherograms was performed using the PAST software package.

### 16S rDNA amplicon sequencing and data handling

To assess precisely the bacterial community composition of the initial biofilm sample as well as of the aerobic and microaerobic enrichments of the 5th week Illumina 16S rDNA amplicon sequencing was carried out. The variable V3 and V4 region of the 16S rDNA was amplified using the primers recommended by Klindworth et al. (2013). PCR amplification was carried out using the KAPA HiFi HotStart Ready Mix (KAPA Biosystems) according to the 16S metagenomics sequencing library preparation guide of Illumina. Paired-end fragment reads were generated on an Illumina MiSeq sequencer using MiSeq Reagent Kit v3 (600-cycle). Read numbers were the following: 195 745 for AER2, 174 767 for MIK1 and 222 988 for initial biofilm sample (BF). Primary data analysis (base-calling) was carried out with Bbcl2fastq^ software (v2.17.1.14, Illumina). Reads were quality and length trimmed in CLC Genomics Workbench Tool 9.5.1 using an error probability of 0.05 (Q13) and a minimum length of 50 nucleotides as threshold. Trimmed sequences were analysed, BLASTN alignment was performed using the most recent SILVA rRNA database. Downstream taxonomical analysis and visualization of the retained reads were performed by the MEGAN6 software (Huson et al. 2007). The 16S Percent Identity Filter item was used as an additional filter for assigning the 16S amplicon reads to a specific taxonomic level. The filter was set to a minimum of 97% for the genus level identification. To avoid sample size effect before comparisons and further analysis, the number of reads was normalized. The 16S rDNA amplicon sequence reads (raw data in FASTQ format) were deposited in the SRA under the study accession number SRP159162 (BioProject PRJNA488537).

### Cloning of *alkB* amplicons, Sanger-sequencing and phylogenetic analysis

The *alkB* PCR amplicons generated with the primer set alkB 1f_deg/alkB 1r_deg were cloned and sequenced (Táncsics et al. 2012) from enrichment cultures AER2 and MIK1 (48 clones in case of each enrichment). The *alkB* sequences of each clone library were manually grouped into operational protein units (OPU) by applying a cutoff value of 0.03. Terminal restriction fragments (T-RFs) predicted in silico for representative clones of each of the OPUs were verified in vitro. Maximum-likelihood phylogenetic tree was reconstructed based on the deduced amino acid sequences using MEGA ver. 7.0 (Kumar et al. 2016). For tree reconstruction, the Jones–Taylor–Thornton model was used, gaps were treated by complete deletion, the number of bootstrap replications was set to 1000, while the substitution rates were set to be the same among the sites and lineages. All of the clone sequences obtained in the present study were deposited with GenBank and can be found under the accession numbers MK390373–MK390468.

### Genome-resolved metagenomics and phylogenomics of organism with novel *alkB*

Metagenome DNA quality and integrity was analysed using an Agilent 2200 Tapestation system. Paired-end fragment reads (2 × 250 nucleotides) were generated using the MiSeq Reagent Kit v2 (500-cycles) with an Illumina MiSeq sequencer. The total cluster number was 8 890 097. Primary data analysis (base-calling) was carried out with “bcl2fastq” software (v.2.17.1.14, Illumina). Quality check of raw reads was performed using BBDuk (v. 37.09; https://sourceforge.net/projects/bbmap/) followed by SICKLE (https://github.com/najoshi/sickle) and subsequently assembled and scaffolded using metaSPADES (v 3.13; Nurk et al. 2017). Genes of scaffolds larger than 1 kb were predicted with prodigal in the meta mode (Hyatt et al. 2010) and annotated using diamond blast (Buchfink et al. 2015) against UniRef100 (Suzek et al. 2007). 16S rRNA gene sequences were predicted as described previously (Brown et al. 2015) and annotated against SILVA 132 (Quast et al. 2013). The taxonomy of the annotated genes was used to calculate a consensus taxonomy for each scaffold (Schulze-Makuch et al. 2018). Coverage of scaffolds was calculated with bowtie2 (mode-sensitive; Langmead and Salzberg 2012).

The novel *alkB* gene from T-RFLP analysis was used to identify the scaffold carrying the respective complete gene (100% identity) and the genome was binned using emergent self-organizing maps (ESOM) based on tetranucleotide frequencies (Dick et al. 2009). The obtained bin was cleaned using GC, coverage and taxonomy of scaffolds. Completeness estimation was based on the presence of 51 bacterial single copy genes.

For community analysis, scaffolds carrying an annotated ribosomal protein S3 (rpS3) were extracted and the coverage of the respective scaffolds was used to calculate a rank abundance curve.

For calculating a phylogenetic tree, 16 ribosomal proteins (L2, L3, L4, L5, L6, L14, L16, L18, L22, L24, S3, S8, S10, S17 and S19) were extracted of the target organism’s genome (Hug et al. 2016), combined with previous datasets as described earlier (Probst et al. 2018) and aligned using MUSCLE (v3.8.31, Edgar 2004). To remove ambiguously aligned terminal regions, the aligned sequences were end-trimmed in the Geneious software (11.0.5). The resulting 16 protein alignments were then concatenated and all organisms, whose sequences spanned less than 50% of the AA positions in the concatenated alignment, were removed. The remaining sequences were used to build a tree using a maximum-likelihood approximation (Price et al. 2010). Visualization was performed in Dendroscope (v. 3.5.10). Average nucleotide identity (ANI) was calculated using EzBioCloud ANI calculator (Yoon et al. 2017).

The metagenome sequence reads (raw data in FASTQ format) were deposited in the SRA under the accession number SRR9332771. The reconstructed genome can be accessed under accession number VIAD00000000 (BioSample accession number SAMN12125306).

## Results and discussion

### Microbial community compositions as revealed by 16S rDNA amplicon sequencing

**Fig. 1:**
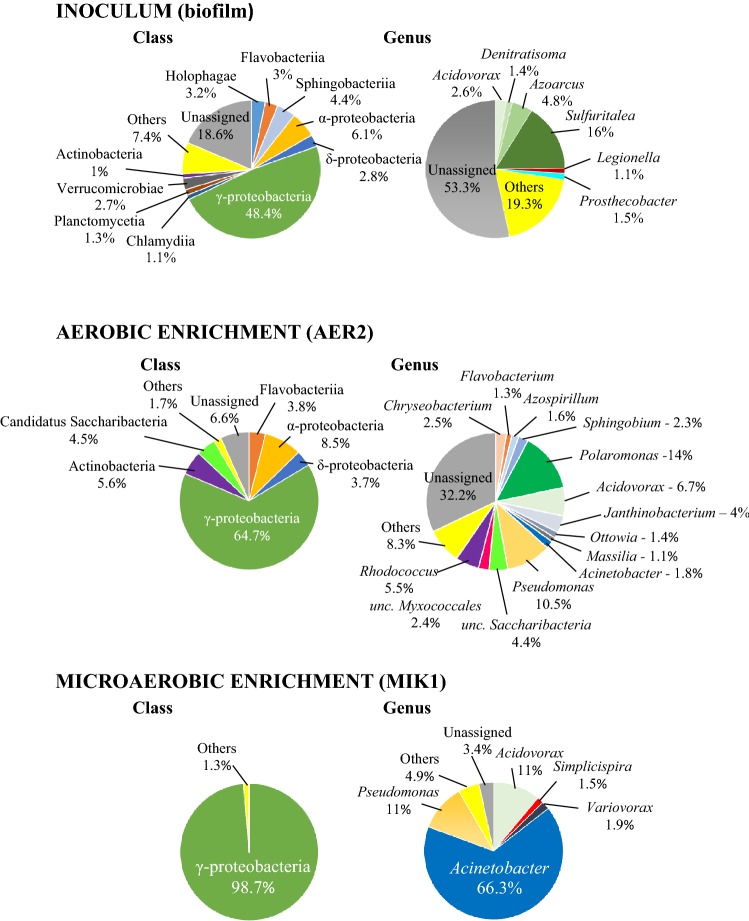
Class and genus level bacterial community structure of the biofilm inoculum, the AER2 aerobic enrichment and, the MIK1 microaerobic enrichment as revealed by Illumina paired-end 16S rDNA amplicon sequencing. All taxa contributing more than 1% abundance were depicted

To reveal how different microbial communities evolve in petroleum hydrocarbon contaminated environments under aerobic and microaerobic conditions, an enrichment approach was used, which proved to be a powerful tool earlier to study the primary effect of oxygen limitation on aromatic hydrocarbon-degrading microbial community structure (Benedek et al. 2018). To establish the enrichment cultures, a biofilm sample was used as inoculum. The biofilm developed in a groundwater monitoring well of a gasoline contaminated site of Hungary, on the stainless steel surface of a submersible pump (Benedek et al. 2016). It was shown earlier that the microbial community of this biofilm has considerably high diversity and was used as inoculum in previous enrichment studies successfully (Benedek et al. 2016, 2018). The new biofilm material contained a microbial community overwhelmingly dominated by Betaproteobacteriales of the Gammaproteobacteria. The most abundant genus was *Sulfuritalea* (16% of total 16S rDNA sequence reads)*,* followed by *Azoarcus* (4.8%), *Acidovorax* (2.6%), *Simplicispira* (0.9%), *Thiobacillus* (0.9%), *Hydrogenophaga* (0.7%), *Thauera* (0.6%), *Zoogloea* (0.6%) and *Rhodoferax* (0.5%) (Fig. 1). Although Alphaproteobacteria and Pseudomonadales-related species of the Gammaproteobacteria were also relatively abundant in the community, the typical petroleum hydrocarbon degraders such as the members of the genera *Novosphingobium*, *Sphingobium* and *Pseudomonas* were detected in considerably low amount (typically lower than 0.05% abundance). Overall, the community composition of the biofilm was highly similar to that of usually observed in petroleum hydrocarbon-contaminated subsurface environments in which hypoxic (oxygen-limited) and/or nitrate-reducing conditions prevail.

Enrichment microbial communities were first subjected to 16S rDNA-based T-RFLP analysis to evaluate the level of similarity between the triplicate enrichments. Cluster analysis of the T-RFLP electropherograms by the Jaccard algorithm showed that microbial communities of clear aerobic (AER) and microaerobic (MIK) enrichments were markedly different and clustered separately (Online Resource 1). The cluster that was prepared using the Bray–Curtis algorithm, which takes into account the abundance of T-RFs as well, showed that microaerobic communities shared a high level of similarity. On the other hand, in case of clear aerobic enrichments only AER2 and AER3 communities were similar, while AER1 showed a bit outlying nature (Online Resource 1). Thus, microbial communities of clear aerobic enrichments showed higher variability than that of the microaerobic enrichments. This phenomenon can be explained by the fact that the aerobic enrichments were subjected to much larger disturbance due to the larger scale aeration. Based on the 16S rDNA T-RFLP results, the microbial communities of enrichments AER2 and MIK1 were analysed by 16S rDNA amplicon sequencing, using Illumina Next-Generation Sequencing Technology (Illumina NGS), which is a suitable tool to gain in-depth knowledge about phylogeny of contaminant-degrading microbial communities (Caporaso et al. 2012; Ławniczak et al. 2016; Szczepaniak et al. 2016).

The microbial community of the aerobic enrichment AER2 was dominated by Betaproteobacteriales (36.5% of sequence reads). The most abundant Betaproteobacteriales-related genus was *Polaromonas* (14%), followed by *Acidovorax* (6.7%) and *Janthinobacterium* (4%) (Fig. 1, Table 1). Several members of the genus *Polaromonas* have already been reported to be able to degrade petroleum hydrocarbons such as benzene and toluene (Sun et al. 2010; Xie et al. 2011) and their dominance was reported in a benzene contaminated aquifer (Aburto et al. 2009). Similarly, members of the genus *Acidovorax* are often reported as dominant members of petroleum hydrocarbon degrading microbial communities (Popp et al. 2006; Daghio et al. 2015), and especially the degradation of phenanthrene and chlorobenzene by *Acidovorax* strains is well documented (Nestler et al. 2007; Singleton et al. 2018). Members of the genus *Janthinobacterium* are often detected in aliphatic hydrocarbon-contaminated environments, but they are primarily considered petroleum hydrocarbon tolerant bacteria rather than degrading ones (Giebler et al. 2013; Pham et al. 2014). At the genus level, *Pseudomonas* proved to be the second most abundant group (10.5%). The role of this genus in petroleum hydrocarbon degradation is well known. Among Alphaproteobacteria (8.5% of sequence reads), the genus *Sphingobium* has to be noted since several members of this genus are involved in petroleum hydrocarbon degradation under aerobic conditions (Révész et al. 2018). Another well-known genus with alkane-degrading species is the *Rhodococcus* (class of Actinobacteria) which members were prominent representatives of the aerobic enrichment community. This was expected since *alkB* genes can be found in almost all members of this genus (Táncsics et al. 2015) and especially some species of the so-called “*erythropolis*” clade are highly efficient aerobic alkane degraders. It is supposed that members of this clade are generalist species of petroleum hydrocarbon contaminated soils, since they are the most commonly detectable hydrocarbon-degrading bacteria in contaminated soils, regardless of soil type or alkane profile of the contaminating petroleum hydrocarbon (e.g. crude oil, diesel or kerosene) (Hamamura et al. 2006, 2013). Some of them encode three or more different *alkB* genes (Whyte et al. 2002), and can produce biosurfactants (typically glycolipids) (Lang and Philp 1998). Interestingly, *Candidatus* Saccharibacteria-related 16S rDNA sequences were also found in relatively high abundance (4.5%). This group was formerly known as Candidate Division TM7, which members can be found in a wide variety of habitats. Until recently, this group has been known solely through 16S rDNA sequences and due to this it is often referred to as “microbial dark matter” (He et al. 2015). Nevertheless, subdivision 3 of Saccharibacteria (TM7-3) was found to be abundant in diesel fuel-contaminated soil (Winsley et al. 2014) and SIP studies revealed the possible role of TM7 bacteria in aerobic degradation of toluene and benzene (Luo et al. 2009; Xie et al. 2011). Last but not least, two genera of the Flavobacteriia, the *Chryseobacterium* and *Flavobacterium* have to be mentioned as notable members of the aerobic enrichment. Both genera contains species which were isolated from petroleum hydrocarbon-contaminated environments (Szoboszlay et al. 2008; Chaudhary and Kim 2018) and especially members of the genus *Flavobacterium* can have a remarkable role in the degradation of petroleum hydrocarbons in contaminated environments (Rahman et al. 2002).

**Table 1:** List of most notable genera (containing cultivable species and showing > 1% abundance) detected in the aerobic versus the microaerobic enrichments and their predictable role in petroleum hydrocarbon degradation

Genus	Abundance of 16S rDNA sequence reads (%)		Documented petroleum hydrocarbon degradation ability	References
	Aerobic enrichment	Microaerobic enrichment		
*Polaromonas*	14	< 0.1	Benzene, toluene, naphthalene, medium-chain alkanes	Sun et al. (2010), Xie et al. 2011), Jeon et al. (2004) and Scheps et al. (2011)
*Pseudomonas*	10.5	11	Alkanes, monoaromatic hydrocarbons, PAHs	Whyte et al. 1997) and van Beilen and Funhoff (2007)
*Acinetobacter*	1.8	66.3	Monoaromatic hydrocarbons, long-chain alkanes	Lal and Khanna (1996), Di Cello et al. 1997) and Tani et al. (2001)
*Acidovorax*	6.7	11	Benzene, phenanthrene, chlorobenzene	Nestler et al. (2007), Aburto and Peimbert (2011) and Singleton et al. (2018)
*Rhodococcus*	5.5	< 0.5	Alkanes, aromatic and heterocyclic compounds, PAHs	Larkin et al. (2005)
*Janthinobacterium*	4	ND	NR	–
*Chryseobacterium*	2.5	ND	Lubricating oil	Saimmai et al. (2012)
*Sphingobium*	2.3	ND	Monoaromatic hydrocarbons, straight-chain alkanes, PAHs	Maeda et al. (2015), Chaudhary et al. (2017) and Révész et al. (2018)
*Variovorax*	< 0.1	1.9	Benzene, phenanthrene	Posman et al. 2017) and Singleton et al. (2018)
*Azospirillum*	1.6	ND	PAHs	Gałązka et al. (2012)
*Simplicispira*	< 0.1	1.5	NR	–
*Ottowia*	1.4	ND	Phenol	Felföldi et al. (2010)
*Flavobacterium*	1.3	< 0.1	Crude oil	Rahman et al. (2002)
*Massilia*	1.1	< 0.1	NR	–

*ND* not detected, *NR* not reported

The microbial community of the microaerobic enrichment MIK1 was considerably simpler than that of the aerobic enrichment. It was overwhelmingly dominated by Gammaproteobacteria (98.8%) with high abundance of Pseudomonadales-related species, while Actinobacteria, Flavobacteriia and Alphaproteobacteria were only minor members of the community (< 0.5% abundance) (Fig. 1). At the genus level, *Acinetobacter*-related sequence reads were the most abundant (66.3%). Members of this genus are frequently reported as prominent alkane and aromatic hydrocarbon degraders under aerobic conditions (Lal and Khanna 1996; Di Cello et al. 1997; Margesin et al. 2003; Czarny et al. 2019) and some of them are adapted to the degradation of long-chain non-branched alkanes (Tani et al. 2001; Rojo 2009). *Acinetobacter* species can be considerably abundant in deep subsurface oil reservoirs, and most probably they are introduced into this environment by injection water (Orphan et al. 2000; Zhao et al. 2012). Oil reservoirs, especially deep subsurface ones are considered oxygen-limited or anaerobic, thus provide excellent environment for studying anaerobic alkane degradation. Nevertheless, shallow subsurface oil reservoirs can receive oxygen by surface recharge of meteoric waters (Jones et al. 2007), while deep subsurface oil reservoirs receive oxygen with the injection water during production, thus niches with trace amounts of oxygen may be present in these environments. Due to these facts, it was assumed that biodegradation of petroleum hydrocarbons in subsurface oil reservoirs is a joint achievement of aerobic and anaerobic microbes (da Cruz et al. 2011). Overall, it can be speculated that members of the genus *Acinetobacter* became dominant in the microaerobic enrichments due to their possible ability to cope with oxygen limitation and outcompeted other aerobic alkane degraders. The second most abundant Gammaproteobacterial genus was the *Pseudomonas* (11%). Recently, it has been reported by Tribelli et al. (2018) that in *P. extremaustralis* strain 14–3^T^ expression of genes involved in alkane degradation (e.g. the *alkB* gene itself) were up-regulated under low-oxygen conditions and the strain was able to use diesel fuel as sole source of carbon under microaerophilic conditions. The possible genetic background of this phenomenon was revealed, and it was shown that similar mechanism may be present in other alkane-degrading *Pseudomonas* strains (Tribelli et al. 2018) and can be important degraders in oxygen-limited petroleum hydrocarbon-contaminated environments. Betaproteobacteriales-related sequence reads belonged mainly to the genera *Acidovorax* (11%), *Variovorax* (1.9%) and *Simplicispira* (1.5%). Some members of the genera *Acidovorax* and *Variovorax* are known aromatic hydrocarbon degraders (Sydow et al. 2016; Posman et al. 2017; Singleton et al. 2018). Moreover, *Acidovorax*-related bacteria can be highly abundant in BTEX-degrading enrichment cultures under hypoxic conditions (Benedek et al. 2018). Although members of the genus *Simplicispira* can be isolated from petroleum hydrocarbon-contaminated subsurface environments (Benedek et al. 2016), and their high abundance was shown in benzene-degrading enrichment cultures under nitrate-reducing conditions (Keller et al. 2018), their role in these environments is still unclear.

The predictable role in petroleum hydrocarbon degradation of the above mentioned, most notable genera (containing cultivable species and showing > 1% abundance) detected either in the aerobic or microaerobic enrichments is summarized in Table 1.

### Diversity and phylogenetic analysis of *alkB* genes in the enrichment cultures

**Fig. 2:**
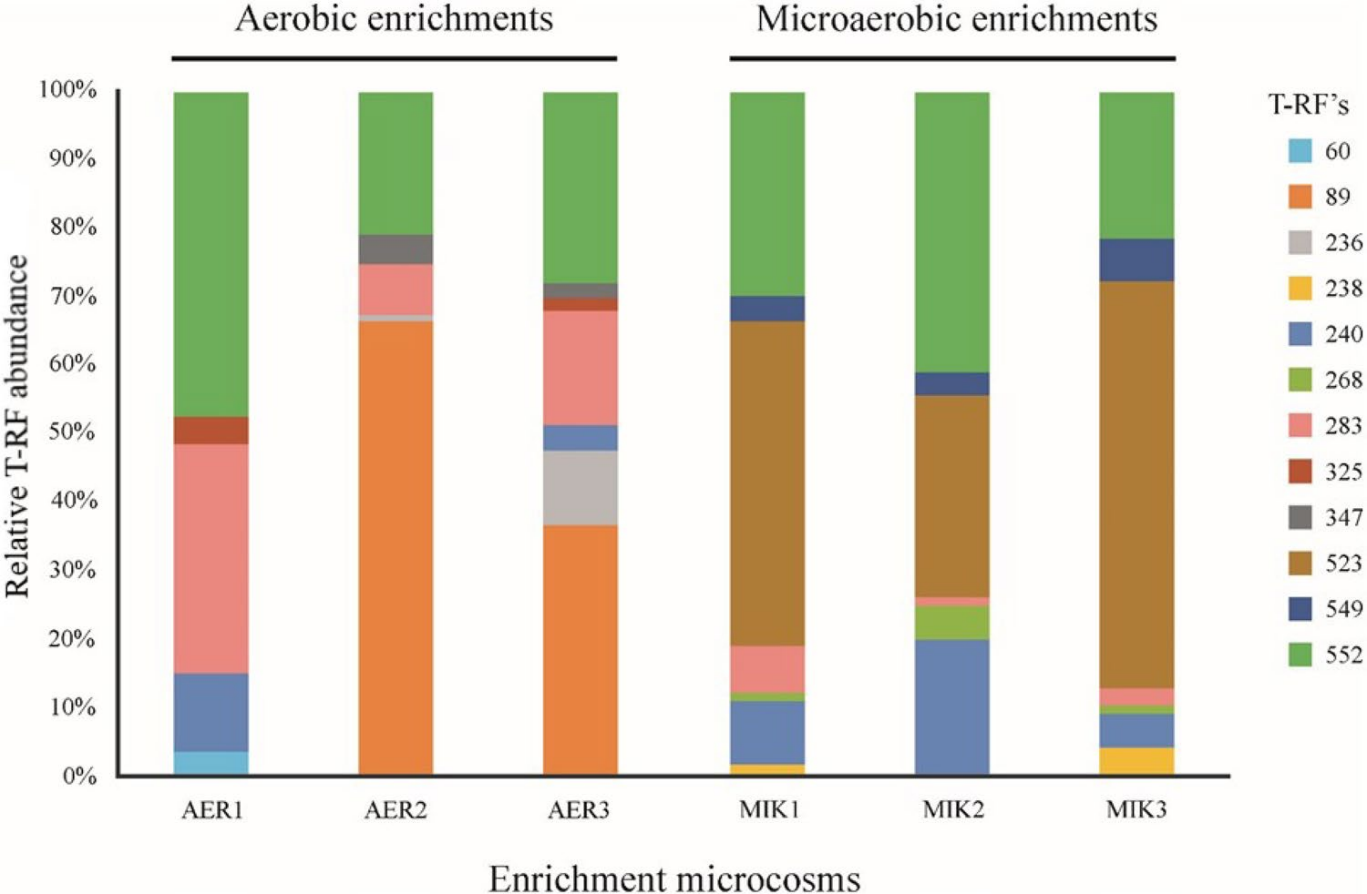
Abundance of *alkB* gene T-RFs in triplicates of the enrichment microcosms at the fifth week of the enrichment procedure. For clone library-based *alkB* gene diversity analysis and for the identification of T-RFs samples, AER2 and MIK1 were used

To reveal and compare *alkB* gene diversity in the aerobic and microaerobic enrichment cultures a T-RFLP assay was used, described by Giebler et al. (2014) as the most appropriate T-RFLP protocol to analyze alkane-degrading bacterial communities. Results of the cluster analysis of the *alkB* gene T-RFLP electropherograms were in accordance with results of the 16S rDNA-based analysis (Online Resource 2). Accordingly, the *alkB* T-RFLP patterns of the parallel microaerobic enrichments were highly similar and could be characterized with the 523-bp long T-RF (Fig. 2). The *alkB* diversity of the aerobic enrichments showed higher variability than that of the microaerobic enrichments. In the enrichments AER2 and AER3, the 89-bp long T-RF was the characteristic T-RF, but it was missing in case of AER1. To link sequence information to the detected T-RFs, *alkB* clone libraries were generated and analysed in case of enrichments AER2 and MIK1.

**Fig. 3:**
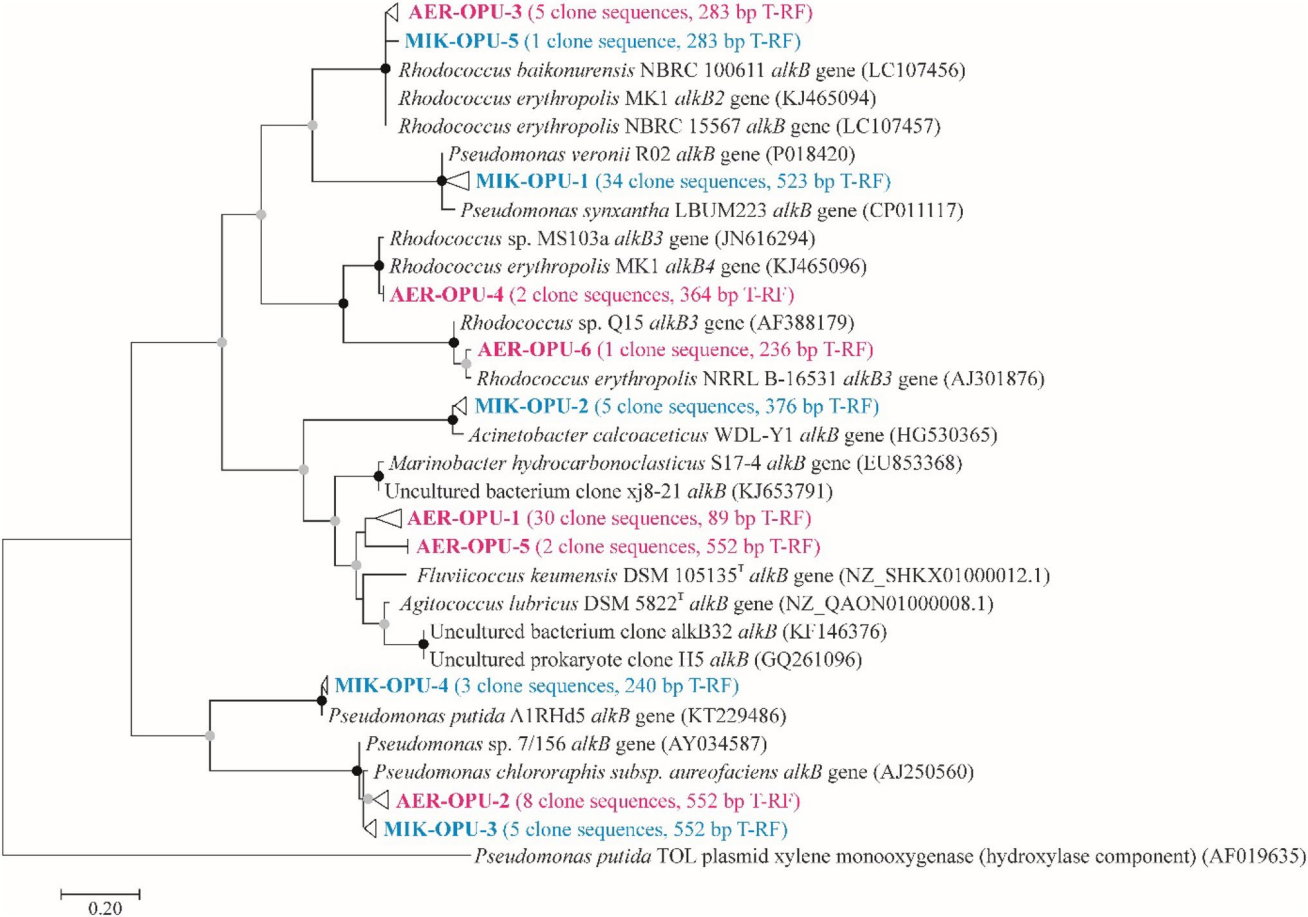
Maximum-likelihood tree showing the phylogenetic position of alkB amino acid sequences retrieved from the aerobic enrichment AER2 (red color) and the microaerobic enrichment MIK1 (blue color). Bootstrap values from 1000 resamplings are indicated with black circles for values of 95–100% and gray circles for values between 50 and 94%. OPUs were determined using a distance cutoff of 0.03 (97% sequence similarity). The tree was rooted with a xylene monooxygenase (hydroxylase component) amino acid sequence of TOL plasmid pDK1 (*Pseudomonas putida*) (color figure online)

In the *alkB* clone library of the aerobic enrichment AER2, the sequences could be divided into six OPUs (Fig. 3). In case of the largest cluster (AER-OPU 1, 63% of clones), sequences showed considerably low similarity to known *alkB* genotypes, therefore, it was not possible to link them to any cultivated bacterium. The most similar nucleotide sequences (with a similarity level of 76–80%) were retrieved from *Agitococcus lubricus* DSM 5822^T^, and from environmental samples, e.g. crude oil-contaminated seawater (Wang et al. 2014) and from soil samples of the sub-Antarctic Macquarie Island (Powell et al. 2010). T-RFLP analysis of individual clones showed that these sequences could be characterized with the 89-bp long T-RF. Most of the other clusters (AER-OPU 2, 3, 4 and 6) contained *Pseudomonas* (*P. putida* and *P. chlororaphis *subsp.* aureofaciens*) and *Rhodococcus*-related *alkB* gene sequences, except AER-OPU 5 which contained sequences most closely related to AER-OPU 1.

In the *alkB* clone library of the microaerobic enrichment MIK1, the sequences could be divided into five OPUs (Fig. 3). The vast majority of sequences (71% of clones, 238/523 bp T-RF) belonged to MIK-OPU 1, and showed high similarity (98.4% at nucleotide level) with *alkB* gene sequences of *Pseudomonas veronii* strains. The closest relative of *P. veronii* is *P. extremaustralis*, which bacterium is capable of degrading aliphatic hydrocarbons under microaerophilic conditions (Tribelli et al. 2018). Although *P. veronii* strains have already been reported to degrade BTEX-compounds and alkyl methyl ketones, still little is known about their role in alkane degradation (Morales et al. 2016; Onaca et al. 2007). Our results indicate that *P. veronii*, similarly to *P. extremaustralis* may also prefer microaerobic conditions for growth on alkanes. Despite to the fact that *Acinetobacter* species were considerably more abundant in enrichment MIK1 than members of the genus *Pseudomonas*, *Acinetobacter*-related *alkB* sequences were found only in low amount (MIK-OPU 2, 10% of clones, 376-bp T-RF) and showed the highest similarity (97.8% at nucleotide level) with *alkB* gene of *A. calcoaceticus* strain CA16. However, it has to be noted here that the PCR primers used in the present study for T-RFLP and cloning purposes do not amplify all types of *alkB* genes encoded by *Acinetobacter* species (Jurelevicius et al. 2013). Moreover, several alkane-degrading *Acinetobacter* strains harbor alkM-type alkane hydroxylases, rather than alkB-type ones. Clone sequences belonging to MIK-OPU 3 and 4 showed high similarity to *Pseudomonas*-related *alkB* sequences (10% and 6% of clone sequences, 548 and 238-bp T-RF). It has to be noted here that clone sequences of MIK-OPU 3 and AER-OPU 2 were identical. The least abundant MIK-OPU 5 contained a single-clone sequence showing similarity to *Rhodococcus*-related *alkB* gene sequences (and to sequences of AER-OPU 3).

### Metagenome sequencing of enrichment AER2: phylogenetic affiliation of the abundant *alkB* genotype by genome binning

**Fig. 4:**
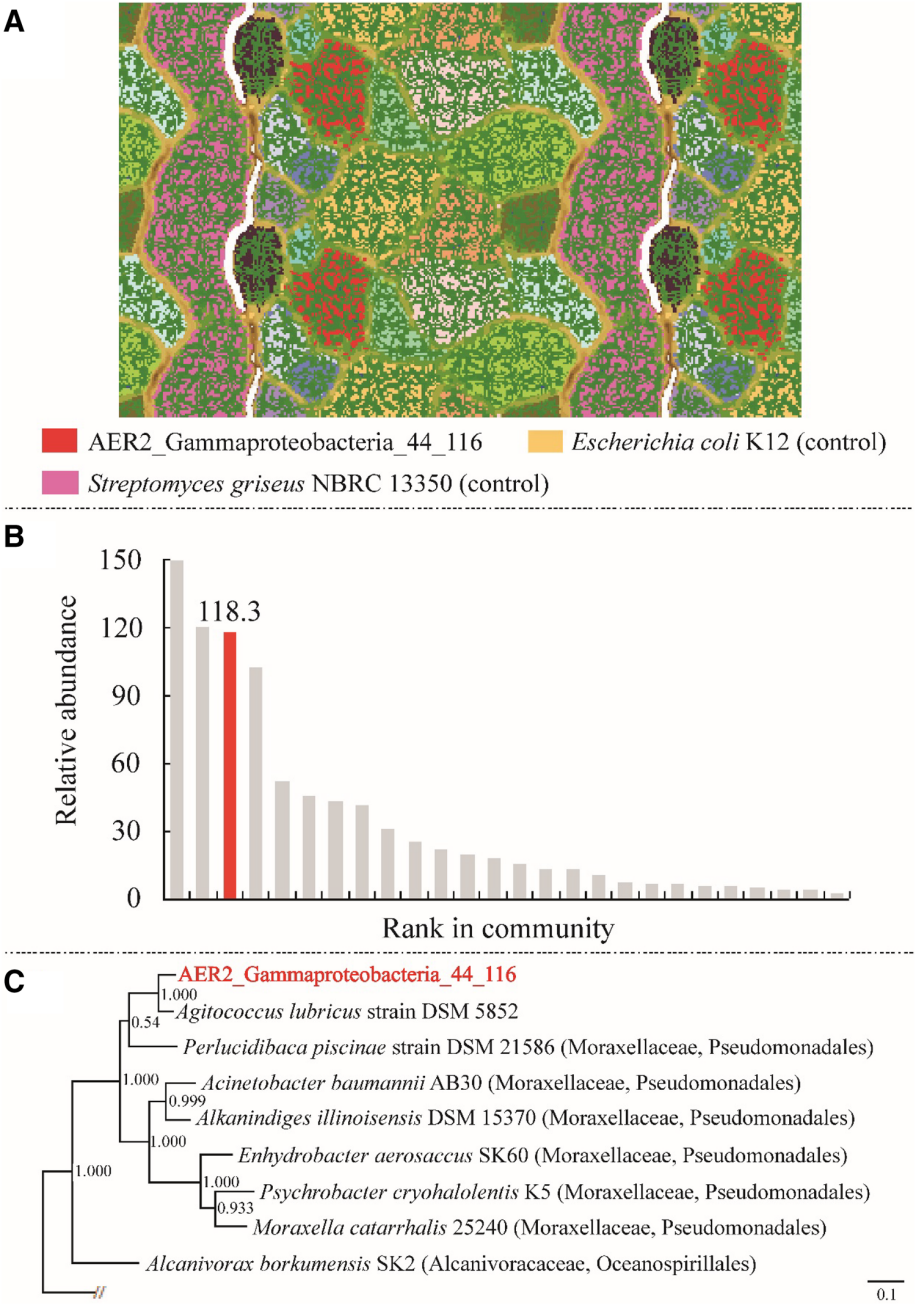
Genome-resolved metagenomics for identification of the genome, to which the novel *alkB* gene from TRFLP analysis belongs. **a** ESOM of sample AER2, highlighting in red the bin carrying the scaffold with the *alkB* gene, named Gammaproteobacteria_44_116 based on its taxonomy, GC, and coverage in the metagenome. *Escherichia coli* K12 and *Streptomyces griseus* NBRC13350, which were used as controls for constructing the ESOM, are also shown. **b** Rank-abundance curve of sample AER2 based on ribosomal protein S3. Red column corresponds to Gammaproteobacteria_44_116. **c** Phylogenetic tree for Gammaproteobacteria_44_116, constructed using 16 concatenated ribosomal proteins. The shown tree is an excerpt of a tree encompassing 3618 genomes, which is provided Online Resource 3. For details, please see methods (color figure online)

**Fig. 5:**
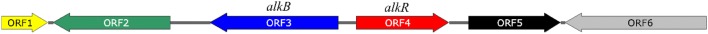
Schematic representation of the *alkB* gene-containing cluster located in the genome of Gammaproteobacteria_44_116. *ORF1* tRNA (cytidine(34)-2′-*O*)-methyltransferase, *ORF2* alpha/beta fold hydrolase, *ORF3* alkane-1 monooxygenase, *ORF4* AraC family transcriptional regulator, *ORF5* short subunit dehydrogenase, *ORF6* oxygen-independent coproporphyrinogen III oxidase. Arrows indicate the orientation of the ORFs

Among the *alkB* genotypes recovered in this study, only those remained unaffiliated, which belonged to AER-OPU 1 and AER-OPU 5. Since AER-OPU 1 contained the most abundant *alkB* gene sequences in case of the aerobic enrichment AER2, genome-resolved metagenomics was used to link them to a bacterial lineage. This novel and abundant *alkB* gene was successfully reconstructed in metagenomic assemblies of the aerobic enrichment culture AER2. The gene was on a scaffold with the length of 554 kb, which was confidentially classified as Gammaproteobacterial based on the consensus taxonomy of 505 predicted and annotated proteins. Using tetranucleotide frequencies, which work reliably well on long scaffolds as given here, we generated an ESOM map (Dick et al. 2009), which is depicted in Fig. 4a. The manually curated genome bin had a GC content of 44%, an average coverage of 116, a completeness of > 99% and 0% detectable contamination. Based on the ribosomal protein S3-based rank abundance curve from the metagenomic data, we provide evidence that the genome belonged to the third most abundant organism in the sample (Fig. 4b). Since no 16S rRNA gene sequence could be binned along with the genome, we investigated the ribosomal proteins of the binned genome. Based on blastp against NR, the organism is closely related to *Agitococcus lubricus* (Franzmann and Skerman 1981), a Firmicute based on NCBI taxonomy, which did not agree with the predicted taxonomy of the bin. To investigate correct placement of the organism and *Agitococcus lubricus* on the tree of life, we generated a phylogenetic tree based on concatenated ribosomal proteins, placing both organisms’ genomes into the clade of *Moraxellaceae* of the Gammaproteobacteria (Fig. 4c). With an ANI of 74.36% over a 1.1 Mbps, the recovered Gammaproteobacteria genome is closely related to *A. lubricus*, however, the organism represents at least a novel species (Kim et al. 2014). *Agitococcus lubricus,* which was misclassified in the NCBI taxonomy, grows on Tween (80, 40 and 20) (Franzmann and Skerman 1981), a complex hydrocarbon, suggesting catabolic similarity between the two organisms. Analysis of the *alkB* gene containing cluster revealed that in upstream position to the *alkB* gene an AraC family transcriptional regulator coding gene can be found (in opposite orientation) (Fig. 5), which had been designated earlier as *alkR* gene in case of *Acinetobacter* sp. strain ADP1. It was found that this gene also plays crucial role in alkane degradation (Ratajczak et al. 1998). Consequently, the combined metagenome-cultivation approach revealed a novel organism, possibly capable of alkane degradation.

## Conclusion and outlook

Overall, the observed differences between the bacterial community compositions of clear aerobic and microaerobic enrichments can be instructive regarding bioremediation of crude oil/diesel fuel-contaminated environments. Results have shown that members of the genus *Rhodococcus* were abundant members of the enrichment communities only under clear aerobic conditions. This observation is important, since rhodococci are frequently used to treat hydrocarbon-contaminated sites due to their enormous metabolic diversity (Kuyukina and Ivshina 2010; Kis et al. 2017). On the other hand, microaerobic conditions caused the overwhelming dominance of *Acinetobacter* and *Pseudomonas* genera-related bacteria. Based on the *alkB* gene diversity analyses, it was also observable that among *Pseudomonas*-related bacteria, the *P. veronii*-lineage was dominant in the microaerobic enrichment cultures. This result together with the fact that the closely related *P. extremaustralis* preferably degrades alkanes under microaerobic conditions enable to assume that a certain group of *Pseudomonas* species is adapted to these conditions and may have an important role in alkane degradation in subsurface ecosystems. Last but not least, a yet unknown but abundant *alkB* genotype was recovered from the aerobic enrichment and was linked to a yet uncultivated member of the family Moraxellaceae by genome binning. Thus, the present study provides new evidence that the known diversity of alkane-degrading bacteria is still incomplete.

## Electronic supplementary material

Below is the link to the electronic supplementary material.

Supplementary material 1 (PDF 338 kb)

Supplementary material 2 (PDF 341 kb)

Supplementary material 3 (DOC 399 kb)
