# Supplementary material for: Microaerobic conditions caused the overwhelming dominance of Acinetobacter spp. and the marginalization of Rhodococcus spp. in diesel fuel/crude oil mixture-amended enrichment cultures
Source: Arch Microbiol. 2019 Oct 29;202(2):329–42. doi: 10.1007/s00203-019-01749-2 (PMC7012980; doi:10.1007/s00203-019-01749-2)
Supplement: Supplementary file 2 — Supplementary material 2 (PDF 341 kb) [file 203_2019_1749_MOESM2_ESM.pdf]

Microaerobic conditions caused the overwhelming dominance of *Acinetobacter* spp. and the marginalization of *Rhodococcus* spp. in diesel fuel/crude oil mixture amended enrichment cultures

Fruzsina Révész[1,2], Perla Abigail Figueroa-Gonzalez[3], Alexander J. Probst[3], Balázs Kriszt[1,2], Sinchan Banerjee[1], Sándor Szoboszlay[2], Gergely Maróti[4], András Táncsics[1,2,*]

[1]Regional University Center of Excellence in Environmental Industry, Szent István University, Gödöllő, Hungary

[2]Department of Environmental Safety and Ecotoxicology, Szent István University, Gödöllő, Hungary

[3]Biofilm Centre, University of Duisburg-Essen, 45141, Essen, Germany

[4]Institute of Plant Biology, Biological, Research Centre of the Hungarian Academy of Sciences, Szeged, Hungary

Journal: Archives of Microbiology

*Corresponding author: András Táncsics, Szent István University, Páter K. u. 1., 2100 Gödöllő, Hungary, tancsics.andras@fh.szie.hu, tel.: 06 28 522 000 #1611

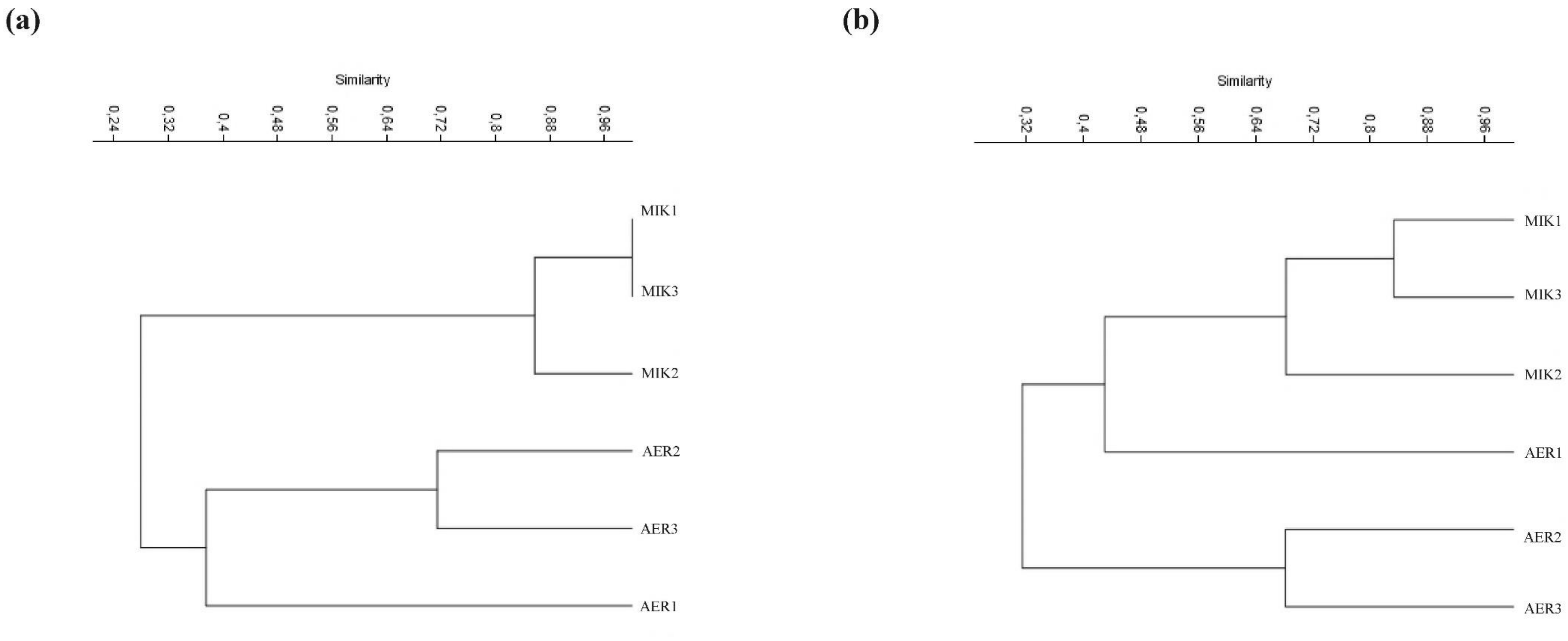

Supplementary Figure 2: Cluster analysis of the *alkB*-based T-RFLP electropherograms by (a) Jaccard and, (b) Bray-Curtis algorithm.
